# Cepharanthine Exerts Antioxidant and Anti-Inflammatory Effects in Lipopolysaccharide (LPS)-Induced Macrophages and DSS-Induced Colitis Mice

**DOI:** 10.3390/molecules28166070

**Published:** 2023-08-15

**Authors:** Guangxin Chen, Da Wen, Lin Shen, Yazhi Feng, Qiuhong Xiong, Ping Li, Zhonghua Zhao

**Affiliations:** 1 State Key Laboratory of Animal Nutrition, College of Animal Science and Technology, China Agricultural University, Beijing 100193, China; 2 State Key Laboratory of Biological Feed, Ministry of Agriculture and Rural Afairs, Boen Biotechnology Co., Ltd., Ganzhou 341000, China; 3 Institute of Biomedical Sciences, Shanxi University, Taiyuan 030006, China; wdbio@outlook.com (D.W.); 19581550438@163.com (L.S.); fyz_99@163.com (Y.F.); qxiong@sxu.edu.cn (Q.X.); pingli@sxu.edu.cn (P.L.)

**Keywords:** inflammatory bowel disease, CEP, NRF2, inflammatory response, oxidative stress

## Abstract

Cepharanthine (CEP), a biscoclaurine alkaloid extracted from *Stephania cepharantha* Hayata, has been widely used for the treatment of various acute and chronic diseases, including leukopenia, and snake bites. Here, our objective was to investigate the anti-oxidative stress and anti-inflammatory response effects of CEP in lipopolysaccharide (LPS)-induced macrophages as well as dextran sulfate sodium (DSS)-induced colitis mice. Our findings demonstrated that supplementation with CEP effectively mitigates body weight loss and elevation of disease activity index (DAI), reduces the malondialdehyde (MDA) content to 2.45 nM/mL while increasing the reduced glutathione (GSH) content to 35.53 μg/mL, inhibits inflammatory response, and maintains proper intestinal epithelium tight junctions in DSS-induced wild type (WT) mice. However, it failed to provide protective effects in DSS-induced transcription factor nuclear factor erythroid 2-related factor 2 (NRF2) knockout (NRF2^−/−^) mice. GSH content decreased to 10.85 μg/10^6^ cells following LPS treatment, whereas supplementation with CEP increased the GSH content to 12.26 μg/10^6^ cells. Moreover, CEP effectively attenuated ROS production in LPS-induced macrophages. Additionally, CEP exhibited inhibitory effects on pro-inflammatory cytokines and mediators in LPS-induced macrophages. Furthermore, we observed that supplementation with CEP promoted the expression of NRF2/heme oxygenase 1 (HO-1)/NADPH quinone oxidoreductase-1 (NQO-1) as well as the phosphorylation of the adenosine monophosphate-activated protein kinase alpha 1 (AMPK-α1)/protein kinase B (AKT)/glycogen synthase kinase-3 beta (GSK-3β) signaling pathway in macrophages while inhibiting the phosphorylation of the extracellular signal-regulated kinase (ERK)/c-Jun N-terminal kinase (JNK), and nuclear factor-kappa B p65 (NF-κB p65) signaling pathway in LPS-induced macrophages. Although CEP did not demonstrate inhibitory effects on oxidative stress or promote the expression of HO-1/NQO-1, it effectively activated the phosphorylation of the AMPK-α1/AKT/GSK-3β signaling pathway which is an upstream regulator of NRF2 in LPS-induced primary peritoneal macrophages from NRF2^−/−^ mice. In summary, our findings suggest that CEP exerts protective effects against oxidative stress and inflammatory response by activating the AMPK-α1/AKT/GSK-3β/NRF2 signaling pathway while concurrently inhibiting the activation of mitogen activated protein kinases (MAPKs) and the NF-κB p65 signaling pathway. These results not only elucidate the mechanisms underlying CEP’s protective effects on colon oxidative stress and inflammation but also provide evidence supporting NRF2 as a potential therapeutic target for IBD treatment.

## 1. Introduction

Inflammatory bowel diseases (IBDs) are chronic, progressive, and relapsing inflammatory disorders that affect the gastrointestinal tract. The two primary types of IBDs are ulcerative colitis (UC) and Crohn’s disease [1,2]. These conditions are characterized by a dysregulated immune response leading to intestinal inflammation and tissue damage [3,4]. The etiology of IBDs is multifactorial, host genetics, microbial factors, environmental factors, overuse of antibiotics, hormone regulation, and chronic immune activation all contribute to their development [3,5]. Although traditional general anti-inflammatory and anti-immunosuppressive drugs, such as 5-aminosalicylates, corticosteroids, and thiopurines have demonstrated efficacy in many IBD patients, the introduction of biologic drugs targeting specific pathways such as the anti-TNF-α, anti-IL-12/23, and Janus kinase (JAK) signaling pathways has provided relief to a subset of patients [6,7]. However, there remains a small proportion of IBD patients who exhibit resistance to all currently available medications. Therefore, it is imperative to explore novel treatment targets and develop new therapeutic agents.

Reactive oxygen species (ROS), generated and released by immune cells, play a crucial role as signaling molecules to enhance the immunological functions of immune cells [8]. An appropriate concentration of ROS contributes to mitogenic response and defense against infections [8], however, excessive release of ROS and related products in the local microenvironment can lead to extensive cellular and molecular damage, initiating an intestinal inflammatory response and tissue destruction [9], ultimately impairing intestinal absorption [10]. The gastrointestinal tract has the most opportunities for interaction with exogenous factors, including food antigens and microbes, compared to any other tissues [11]. Although the gastrointestinal tract possesses a robust immune system to maintain host health, consistent exposure to extraneous stimulation could exacerbate the immune system’s response, resulting in overproduction of free radicals or weakening of the endogenous antioxidant system [12]. The imbalance between free radicals and antioxidant systems is commonly referred to as oxidative stress. It represents an imbalance between pro-oxidation and anti-oxidation processes that are associated with inflammatory responses. To date, increasing clinical and experimental evidence has demonstrated that oxidative stress is implicated in the pathogenesis of IBDs [13,14]. Therefore, targeting intestinal tissue oxidative stress may offer potential therapeutic benefits for treating IBD.

Nuclear factor erythroid 2-related factor 2 (NRF2) belongs to the cap ‘n’ collar transcription factor family [15]. It is activated by various stimuli, including oxidative, electrophilic, proteotoxic stress, and small natural molecules. NRF2 plays a pivotal role in safeguarding and restoring cellular homeostasis [16]. Previous studies have demonstrated that NRF2 activation maintains cellular redox homeostasis and regulates inflammatory response in neurodegenerative disease [17], diabetes [16], and kidney disease [18]. In fact, NRF2 activation has exhibited promising outcomes against lung, liver, eye, gastrointestinal, metabolic, neurodegenerative as well as autoimmune diseases where oxidative stress and inflammation contribute to pathogenesis [19]. Pharmacological agents targeting NRF2 such as dimethyl fumarate have been employed for treating multiple sclerosis and psoriasis, and bardoxolone methyl has entered clinical phase II/III trials for treating CTD-PAH, pulmonary hypertension-ILD, Alport syndrome, and autosomal dominant polycystic kidney disease [19]. The protective effects of pharmacological NRF2 activators have been observed in numerous human disease models with benefits seen in human intervention trials. Therefore, NRF2 is considered a suitable drug target for screening against oxidative stress and inflammation during various disease pathogeneses [19].

Previous studies have demonstrated the anti-oxidative property of various natural products, including curcumin analogues and extracts from *isodon suzhouensis*, which provide in vivo and in vitro antioxidant effects [20,21,22]. In this study, we aim to investigate the impact of CEP on oxidative stress and inflammatory response in macrophages and mice. CEP is a biscoclaurine alkaloid derived from *Stephania cepharantha* Hayata that exhibits diverse biological properties. It effectively ameliorates leukopenia and immune thrombocytopenic purpura by maintaining proper leukocyte levels [23,24]. Moreover, it alleviates inflammation and pain caused by snake bites [25]. Additionally, CEP demonstrates various pharmacological activities including anti-inflammatory response through a reduction in NLRP3 inflammasome activation [26] and the inhibition of MAPKs and NF-κB p65 signaling pathway [27,28], antioxidant effects to combat oxidative stress [26], prevention of oxidative stress-induced DNA damage [29], inhibition of autophagy by blocking autophagosome-lysosome fusion and lysosomal cathepsin B/D maturation [30], as well as antiviral activity when combined with nelfinavir to reduce viral load accumulation and facilitate virus elimination [31]. Furthermore, CEP enhances the sensitivity of host or cells to anticancer agents [30,32], although it does not directly affect cancer itself. Intestinal inflammation, oxidative stress, and destruction of intestinal epithelial barrier are well-known symptoms associated with IBD. Notably, CEP possesses inhibitory effects on the inflammatory response and oxidative stress. However, the impact of CEP on IBD remains understudied. Therefore, our objective is to explore the effects of CEP on oxidative stress and the inflammatory response in LPS-induced macrophages as well as DSS-induced colitis mice.

## 2. Results

### 2.1. CEP Ameliorates IBD Characteristics in DSS-Induced Colitis Mice

**Figure 1:**
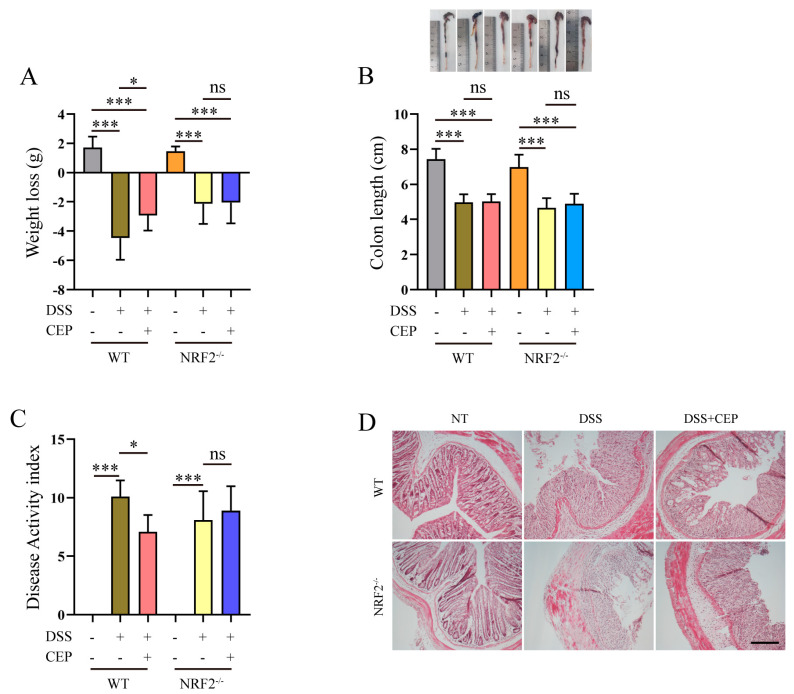
The ameliorative effects of CEP on DSS-induced colitis mice. WT and NRF2^−/−^ mice were pre-treated with CEP for one week followed by treatment with DSS for an additional week. (**A**) The weight loss (initial body weight minus trial termination body weight) of DSS- and CEP-treated WT and NRF2^−/−^ mice, “ns” representative no significance (*n* = 8–12). (**B**) The colon length of DSS- and CEP-treated WT and NRF2^−/−^ mice, “ns” representative no significance (*n* = 8–12). (**C**) The disease activity index (DAI) of DSS- and CEP-treated WT and NRF2^−/−^ mice, “ns” representative no significance (*n* = 8–12). (**D**) H&E staining was performed on colon tissue from DSS- and CEP-treated WT and NRF2^−/−^ mice, magnification shown is 10×, and the scale bar represents 200 μm (*n* = 3–5). The results shown are means ± SEM, * *p* < 0.05, and *** *p* < 0.001.

The essential features of IBD encompass a diminished survival rate, increased body weight loss, shortened colon length, and an elevated clinical score. To investigate the potential role of CEP in colitis, we assessed these crucial characteristic indices in DSS-induced WT and NRF2^−/−^ mice. Pre-treatment with CEP ameliorated weight loss in DSS-induced WT mice, however, this effect was not observed in NRF2^−/−^ mice (Figure 1A). While DSS dramatically reduced colon length in both WT and NRF2^−/−^ mice, supplementation with CEP failed to mitigate this parameter (Figure 1B). Furthermore, DSS augmented the disease activity index (DAI) in both WT and NRF2^−/−^ mice. Nevertheless, supplementation with CEP significantly decreased the DAI only in WT mice but not in NRF2^−/−^ mice (Figure 1C). Histopathological examination evaluated the severity of inflammation and ulceration in colitis-afflicted rodents. Crypt loss, mucosal layer destruction, and edema were exacerbated both in DSS-induced WT and NRF2^−/−^ mice. Conversely, pre-treatment with CEP effectively alleviated intestinal tissue damage only in DSS-induced WT mice, no effects on NRF2^−/−^ mice were observed (Figure 1D).

### 2.2. CEP Inhibits Oxidative Stress and Inflammatory Response in DSS-Induced Colitis Mice

**Figure 2:**
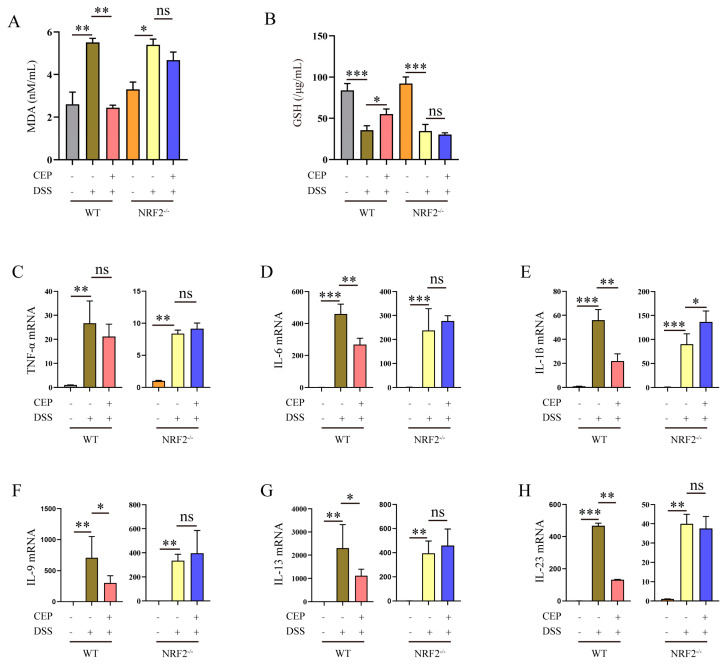
CEP inhibits oxidative stress and inflammatory response in DSS-induced colitis mice. (**A**) The MDA level in serum from CEP and DSS treatment WT and NRF2^−/−^ mice, “ns” representative no significance (*n* = 3). (**B**) The GSH level in serum from CEP and DSS treatment WT and NRF2^−/−^ mice, “ns” representative no significance (*n* = 3). (**C**–**H**). qRT-PCR analyzed the expression of the pro-inflammatory cytokines TNF-α, IL-6, IL-1β, IL-9, IL-13, and IL-23 in colon tissue homogenate, “ns” representative no significance (*n* = 3). The results shown are means ± SEM, * *p* < 0.05, ** *p* < 0.01, and *** *p* < 0.001.

Currently, mounting clinical and experimental evidence strongly supports the involvement of oxidative stress in the pathogenesis of colitis. Therefore, we initially assessed serum oxidative stress levels in DSS-induced mice. Our results demonstrated that DSS treatment significantly increased MDA levels to 5.51 nM/mL in serum from WT mice, while pre-treatment with CEP dramatically reduced MDA level to 2.45 nM/mL in WT mice, however it had no impact on NRF2^−/−^ mice (Figure 2A). Next, we evaluated change in GSH content in DSS-induced WT and NRF2^−/−^ mice. DSS markedly decreased GSH content to 35.53 μg/mL in the serum of WT mice, whereas CEP significantly increased the GSH level to 55.02 μg/mL in WT mice; however, it also had no effect on NRF2^−/−^ mice (Figure 2B). Inflammatory response is a prominent characteristic of colitis, therefore, pro-inflammatory cytokines were measured in DSS-induced WT and NRF2^−/−^ mice. Our findings showed that DSS increased the expression of pro-inflammatory cytokines TNF-α, IL-1β, IL-6, IL-9, IL-13, and IL-23 in both WT and NRF2^−/−^ mice. Pre-treatment with CEP inhibited the expression of pro-inflammatory cytokines in DSS-induced mice but failed to inhibit their rise in DSS-induced NRF2^−/−^ mice (Figure 2C–H).

### 2.3. CEP Maintains Proper Intestinal Epithelium Barrier in DSS-Induced Colitis Mice

**Figure 3:**
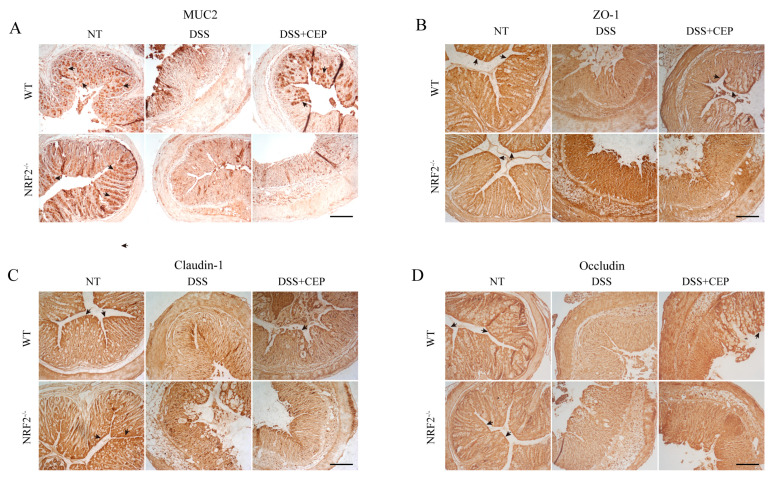
CEP maintains proper intestinal epithelium barrier in DSS-induced colitis mice. (**A**) Immunohistochemistry analysis was performed to evaluate the expression of MUC2 in the colon tissue from DSS- and CEP-treated WT and NRF2^−/−^ mice treated with DSS and CEP, magnification shown is 10×, the scale bar represents 200 μm, and the arrow marked the location and abundance of the protein (*n* = 3–5). (**B**–**D**) Immunohistochemistry analysis was conducted to assess the expression of ZO-1 (**B**), Claudin-1 (**C**), and Occludin (**D**) in the colon tissue from DSS- and CEP-treated WT and NRF2^−/−^ mice, magnification shown is 10×, the scale bar represents 200 μm, and the arrow marked the location and abundance of the protein (*n* = 3–5).

Immunohistochemistry analysis revealed a decrease in mucin 2 (MUC2) protein expression in both DSS-induced WT and NRF2^−/−^ mice. In WT mice, CEP promoted the expression of MUC2 in colon tissue, while no effects were observed in NRF2^−/−^ mice (Figure 3A). Tight junction proteins play a crucial role in maintaining the integrity of the intestinal epithelial barrier. Therefore, we evaluated the levels of ZO-1, Claudin-1, and Occludin in colon tissue from WT and NRF2^−/−^ mice. Immunohistochemistry results demonstrated that DSS significantly disrupted the structure of colon tissue and reduced the expression of ZO-1, Claudin-1, and Occludin in both DSS-induced WT and NRF2^−/−^ mice. However, pre-treatment with CEP effectively mitigated the colon tissue damage by restoring its structural integrity and enhancing the expression of ZO-1, Claudin-1, and Occludin specifically in WT mice but had no effect on NRF2^−/−^ mice.

### 2.4. CEP Inhibits Inflammatory Response and Oxidative Stress in LPS-Induced Macrophages

**Figure 4:**
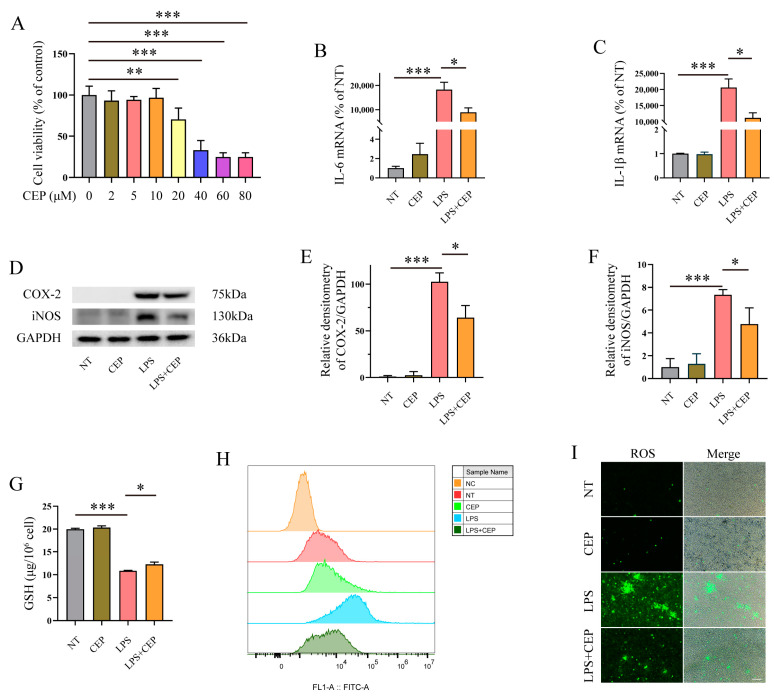
CEP inhibits inflammatory response and oxidative stress in LPS-induced macrophages. (**A**) The CCK8 assay analyzed the impact of CEP on macrophage viability (*n* = 5). (**B**,**C**) qRT-CPR analyzed the expression of the pro-inflammatory cytokines IL-6 (**B**) and IL-1β (**C**) in LPS and CEP treated macrophages (*n* = 3). (**D**–**F**) Western blot analysis of the impact of CEP on the expression of COX-2 (**E**) and iNOS (**F**) in LPS-induced macrophages (*n* = 3). (**G**) The GSH level in the medium of CEP and LPS induced-macrophages (*n* = 3). (**H**,**I**) Flow cytometry and cell fluorescence analyzed the production of ROS in LPS-induced macrophages (*n* = 3). The results shown are means ± SEM, * *p* < 0.05, ** *p* < 0.01, and *** *p* < 0.001.

Next, we investigated the impact of CEP on inflammatory response and oxidative stress in LPS-induced macrophages. The concentration of CEP (20 μM) for macrophage treatment was determined based on the results obtained from the CCK8 assay (Figure 4A). qRT-PCR analysis revealed a significant inhibition of IL-6 and IL-1β expression in LPS-induced macrophages following pre-treatment with CEP (Figure 4B,C). Western blot analysis demonstrated that COX-2 and iNOS expression were suppressed by pre-treatment with CEP in LPS-induced macrophages (Figure 4D–F). These findings indicate that supplementation with CEP effectively attenuates the inflammatory response in LPS-induced macrophages. Furthermore, we evaluated the effects of CEP on oxidative stress. Our results showed that GSH levels were significantly reduced to 10.85 μg/10^6^ cells upon exposure to LPS, while supplementation with CEP increased it to 12.26 μg/10^6^ cells in macrophages (Figure 4G). Flow cytometry and cell fluorescence data indicated a substantial increase in ROS levels upon exposure to LPS, which were dramatically decreased by treatment with CEP in macrophages (Figure 4H,I). 

### 2.5. The Effects of CEP on NRF2/HO-1/NQO-1, and AMPK-α1/AKT/GSK-3β Signaling Pathways

**Figure 5:**
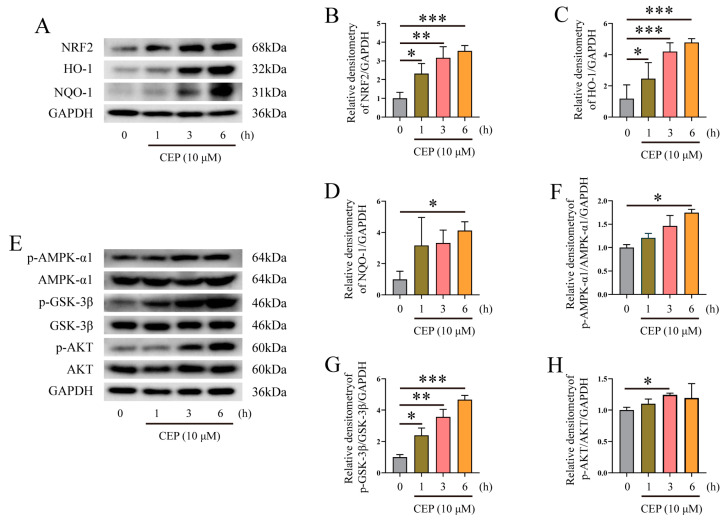
The effects of CEP on the NRF2/HO-1/NQO-1, and AMPK-α1/AKT/GSK-3β signaling pathways. (**A**–**D**) Western blot analysis of the impact of CEP on the expression of NRF2 (**B**), HO-1 (**C**), and NQO-1 (**D**) signaling pathway in LPS-induced macrophages (*n* = 3). (**E**–**H**) Western blot analysis of the impact of CEP on the phosphorylation of the AMPK-α1 (**F**), AKT (**G**), and GSK-3β (**H**) signaling pathways in LPS-induced macrophages (*n* = 3). The results shown are means ± SEM, * *p* < 0.05, ** *p* < 0.01, and *** *p* < 0.001.

NRF2, is the most crucial transcription factor against oxidative stress and plays a pivotal role in anti-oxidants and inflammation relief [33]. Therefore, we investigated the effects of CEP on NRF2 and its related signaling pathway. The results indicated that treatment with CEP promoted the expression of NRF2/HO-1/NQO-1 in a time-dependent manner (Figure 5A–D), suggesting that CEP may possess anti-oxidative properties. Previous studies have demonstrated that GSK-3β is a novel regulator of NRF2, therefore, the anti-oxidative property of NRF2 may be associated with the phosphorylation of the AMPK-α1/AKT/GSK-3β signaling pathway [34]. Consequently, we explored the impact of CEP on the AMPK-α1/AKT/GSK-3β signaling pathway in macrophages. Our findings revealed that CEP enhanced the phosphorylation of the AMPK-α1/AKT/GSK-3β signaling pathway (Figure 5E–H).

### 2.6. CEP Inhibits the Phosphorylation of MAPKs and the NF-κB Signaling Pathway

**Figure 6:**
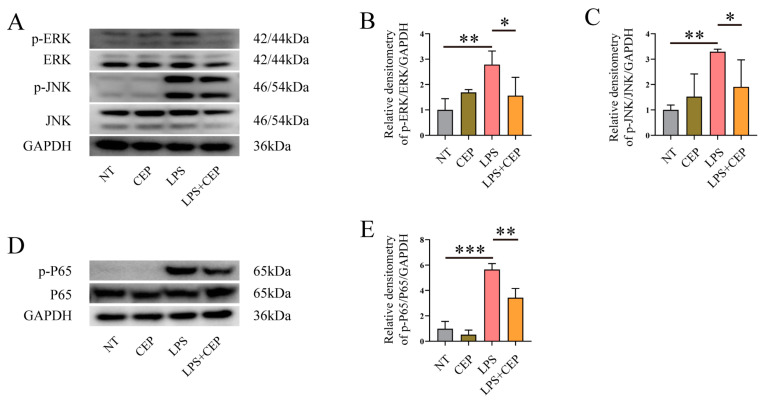
CEP inhibits the phosphorylation of MAPKs and the NF-κB signaling pathway. (**A**–**C**) Western blot analysis of the impact of CEP on the phosphorylation of ERK (**B**), and JNK (**C**) signaling pathways in LPS-induced macrophages (*n* = 3). (**D**,**E**) Western blot analysis of the impact of CEP on the phosphorylation of the NF-κB p65 signaling pathway in LPS-induced macrophages (*n* = 3). The results shown are means ± SEM, * *p* < 0.05, ** *p* < 0.01, and *** *p* < 0.001.

The Western blot results revealed a significant upregulation of the ERK and JNK signaling pathway phosphorylation upon LPS stimulation, whereas pretreatment with CEP effectively attenuated the phosphorylation effects (Figure 6A–C). Additionally, CEP exhibited inhibitory effects on the NF-κB p65 signaling pathway in LPS-induced macrophages (Figure 6D,E).

### 2.7. The Effects of CEP on Oxidative Stress in LPS-Induced NRF2^−/−^ Mice Primary Peritoneal Macrophages

**Figure 7:**
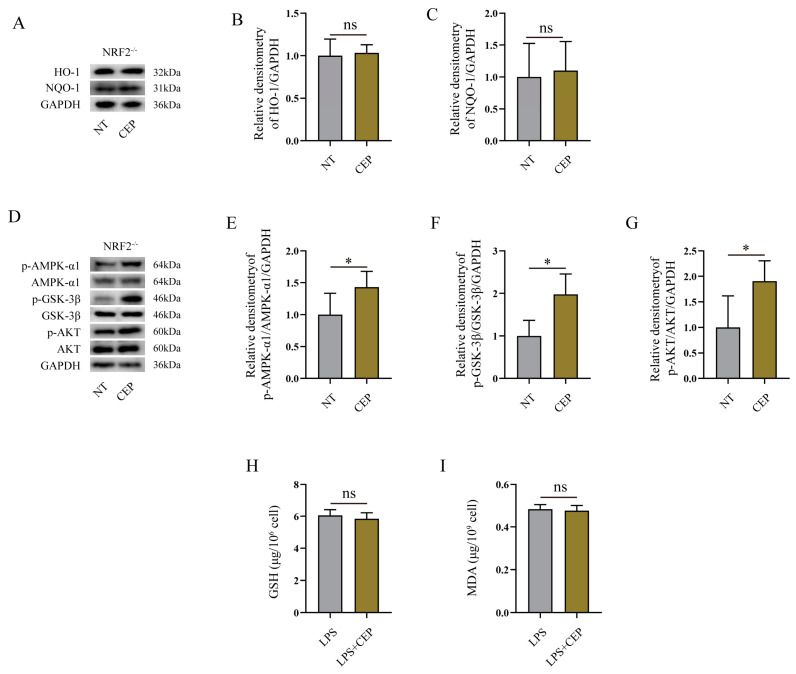
The effects of CEP on oxidative stress in LPS-induced NRF2^−/−^ mice primary peritoneal macrophages. (**A**–**C**) Western blot analysis of the impact of CEP on the expression of HO-1 (**B**), and NQO-1 (**C**) signaling pathway in primary peritoneal macrophages from NRF2^−/−^ mice, “ns” representative no significance (*n* = 3). (**D**–**G**) Western blot analysis of the impact of CEP on the phosphorylation of AMPK-α1, GSK-3β, and AKT signaling pathways in primary peritoneal macrophages from NRF2^−/−^ mice (*n* = 3). (**H**) The GSH level in medium of CEP and LPS induced-primary peritoneal macrophages from NRF2^−/−^ mice, “ns” representative no significance (*n* = 3). (**I**) The MDA level in medium of CEP and LPS induced-primary peritoneal macrophages from NRF2^−/−^ mice, “ns” representative no significance, (*n* = 3). The results shown are means ± SEM, * *p* < 0.05.

Our above findings have demonstrated that CEP exerts a protective role against oxidative stress and inflammatory response by activating NRF2 and its related signaling pathway. To further elucidate the underlying mechanism of CEP, we measured its effects on LPS-induced macrophages derived from NRF2^−/−^ mice. Western blot analysis revealed that while CEP failed to enhance the expression of the HO-1/NQO-1 signaling pathway in these cells (Figure 7A–C), it did promote the phosphorylation of the AMPK-α1/AKT/GSK-3β signaling pathway (Figure 7D–G). These results suggest that CEP activates the phosphorylation of the AMPK-α1/AKT/GSK-3β signaling pathway, subsequently facilitating NRF2 activation, and thereby inhibiting oxidative stress in macrophages. To further validate these observations, we evaluated the impact of CEP on LPS-induced primary peritoneal macrophages obtained from NRF2^−/−^ mice. Our data indicated that CEP failed to suppress MDA production and had no effect on the reduced GSH levels in LPS-induced primary peritoneal macrophages obtained from NRF2^−/−^ mice (Figure 7H,I). Collectively, our experiments revealed that although CEP activates the phosphorylation of the AMPK-α1/AKT/GSK-3β signaling pathway, it is evident that NRF2 plays a pivotal role in protecting against oxidative stress in macrophages.

## 3. Discussion

IBD is a highly prevalent chronic gastrointestinal disease globally, and is characterized by a complex pathogenesis involving various factors such as host genetics, intestinal microbiota, environmental triggers, lifestyle factors, and diet [35]. The interaction among these multiple components disrupts the balance within the intestinal mucosa while also altering immune responses [36], leading to an increase in permeability of the intestinal epithelium. The invasion of enterocoel through the epithelial layer triggers immune cell activation and induces the production of pro-inflammatory cytokines. Chronic local inflammation further enhances the generation and release of ROS by immune cells, which serve as crucial signaling molecules in immunological functions [8]. However, excessive production of ROS and related products in the actively inflamed mucosal microenvironment leads to collateral damage, including intestinal inflammation and tissue destruction [9]. Accumulating evidence suggests that oxidative stress plays a pivotal role in the pathogenesis of IBD. Therefore, reducing ROS levels and inhibiting oxidant stress may have significant therapeutic implications for IBD.

CEP is a biscoclaurine alkaloid derived from *Stephania cepharantha* Hayata, which has been extensively utilized in Japan for over 40 years for the treatment of acute and chronic diseases [37]. CEP exhibits diverse pharmacological activities, including anti-inflammatory effects by reducing NLRP3 inflammasome activation [26] and inhibiting MAPKs and NF-κB p65 signaling pathways [27,28], as well as anti-oxidative stress, anti-autophagy, and anti-viral effects [26,30,31]. Furthermore, CEP enhances the sensitivity of host cells to anti-cancer agents [30,32], without exerting any direct impact on cancer itself. The objective of this study is to investigate the effects of CEP on oxidative stress and inflammatory response both in vivo and in vitro.

The essential features of IBD include a depressed survival rate, increased body weight loss, shortened colon length, and elevated clinical score [38]. Our findings demonstrated that supplementation with CEP significantly improves weight loss, DAI, and colon tissue impairment in DSS-induced WT mice but not in NRF2^−/−^ mice. This is consistent with previous studies showing that CEP inhibits the increased weight loss, rise of DAI and histological score in DSS-induced WT colitis mice [39,40]. Transient middle cerebral artery occlusion (tMCAO) increases levels of ROS and MDA, while decreasing SOD levels in mice. However, supplementation with CEP significantly ameliorates oxidative stress in the tMCAO-induced mouse model [26]. Furthermore, CEP inhibits inflammatory response in LPS-induced acute lung injury and the DSS-induced colitis mouse model [28,39]. In this study, we found that supplementation with CEP inhibits oxidative stress and inflammatory response in DSS-induced WT mice, but not in NRF2^−/−^ mice. Additionally, we found that supplementation with CEP maintains proper intestinal epithelium barrier in DSS-induced WT mice. Our results demonstrated that CEP provides a protective role against oxidative stress and inflammatory response in DSS-induced mice. Moreover, CEP had no significant effects on the characteristics observed in DSS-induced NRF2^−/−^ colitis mice, suggesting that the protective functions of CEP on oxidative stress and inflammatory response are in a NRF2-dependent manner.

To further investigate the effects of CEP on oxidative stress and inflammatory response, we assessed its impact on LPS-induced inflammatory response and oxidative stress in macrophages. Our findings demonstrated that 10 μM CEP effectively attenuates the expression of pro-inflammatory cytokines IL-6 and IL-1β, as well as pro-inflammatory mediators iNOS and COX-2 in LPS-stimulated macrophages. Moreover, supplementation with CEP was observed to enhance the reduced GSH level while reducing ROS production in LPS-treated macrophages. These results are consistent with previous studies highlighting the potent anti-inflammatory and anti-oxidant properties of CEP [28,41]. Collectively, our findings along with prior research, provide compelling evidence for the inhibitory effects of CEP on both inflammatory responses and oxidative stress.

Next, we explored the potential mechanism of CEP on oxidative stress and inflammatory response. NRF2 is a pivotal transcription factor that plays a crucial role in combating oxidative stress and inflammatory response [33]. Therefore, we examined the impact of CEP on NRF2 and its associated signaling pathway. Our results demonstrated that supplementation with CEP significantly upregulated the expression of NRF2/HO-1/NQO-1 in a time-dependent manner, indicating the essential role of NRF2 in mediating the anti-oxidative and anti-inflammatory effects of CEP. AMPK activation regulates both catabolism and anabolism, while also maintaining redox balance and modulating inflammatory response [42,43]. Moreover, AMPK promotes phosphorylation events involving PI3K/AKT [44] and GSK-3β, which inhibits mitochondrial oxidative stress [45]. Previous studies have suggested GSK-3β as a novel regulator of NRF2, therefore, it is plausible that the anti-oxidative properties of NRF2 are associated with the involvement of phosphorylation events mediated by the AMPK-α1/AKT/GSK-3β signaling pathway [34]. Our findings revealed that CEP enhanced phosphorylation levels within the AMPK-α1/AKT/GSK-3β signaling pathway. Overall, our study suggests that CEP exerts potent anti-oxidative effects in macrophages through activation of the AMPK-α1/AKT/GSK-3β/NRF2 signaling pathway.

Although activation of NRF2 is generally believed to counter inflammation in various inflammatory diseases [46], natural products may also have impacts on other signaling pathways. Our results demonstrated that supplementation with CEP significantly inhibits the phosphorylation of the ERK, JNK, and NF-κB p65 signaling pathways in LPS-treated macrophages. This is consistent with previous studies showing that CEP inhibits the activation of the MAPKs and NR-κB p65 signaling pathways [28,47,48]. These findings suggest that the effects of CEP on inflammatory response are not limited to NRF2 but also depend on its inhibition of the ERK, JNK and NF-κB P65 signaling pathways. 

Our findings in mice demonstrated that CEP fails to inhibit the oxidative stress and inflammatory response in NRF2-deficient mice, suggesting the crucial role of NRF2 in mediating its effects on anti-inflammatory response and oxidative stress. To validate these results, we investigated the impact of CEP on primary peritoneal macrophages from NRF2^−/−^ mice. The outcomes revealed that CEP failed to enhance the expression of the HO-1/NQO-1 signaling pathway; however, it activated the phosphorylation of the AMPK-α1/AKT/GSK-3β signaling pathway in primary peritoneal macrophages from NRF2^−/−^ mice. Moreover, CEP had no influence on MDA levels and reduced GSH levels in LPS-induced primary peritoneal macrophages from NRF2^−/−^ mice. These findings suggest that while NRF2 plays a significant role in the antioxidant activity of CEP, it also activates the phosphorylation of the AMPK-α1/AKT/GSK-3β pathway which is an upstream regulator of NRF2.

## 4. Materials and Methods

### 4.1. Animal Model

WT and NRF2^−/−^ C57BL/6 mice aged 6–8 weeks-old were utilized in this study. All mice were housed under controlled conditions at a temperature of 22–23 °C on a 12 h light/dark cycle, with ad libitum access to food and water. The mice were randomly divided into six groups, each containing 8–12 individuals: NT group; DSS group, where the animals received a solution of 3% (*w*/*v*) DSS via drinking water for one week; DSS + CEP group, where the mice were administered CEP through intragastric gavage at a dosage of 10 mg/kg, based on previous research [48], followed by DSS treatment for another week. All experimental procedures involving animal subjects have been approved by the Committee of Scientific Research at Shanxi University.

### 4.2. Disease Activity Index

Throughout the experiment of DSS-induced colitis, mice were closely monitored for changes in body weight, stool consistency, and fecal occult blood. Disease activity index (DAI) scores were determined using a previously established scoring system [49].

### 4.3. Hematoxylin and Eosin (H&E) Staining

The colon tissue samples obtained from mice were washed with phosphate-buffered saline (PBS), fixed in a solution containing 4% paraformaldehyde, embedded in paraffin, sectioned into 5 μM slices, and subsequently stained with hematoxylin and eosin. 

### 4.4. Cell Culture

The murine macrophage cell line RAW264.7 was obtained from BeNa Culture Collection and cultured in Dulbecco’s modified Eagle’s medium (Gibco, Grand Island, NY, USA) supplemented with 10% fetal bovine serum (Sorfa, Beijng, China). The cells were incubated at a temperature of 37 °C with 5% CO_2_ in a humidified chamber.

### 4.5. Cell Viability Assay

RAW264.7 macrophages were seeded in 96-well plates and pre-treated with various concentrations of CEP (2, 5, 10, 20, 40, 60, and 80 μM) for a duration of 24 h. Following the incubation, cck8 solution (1 mg/mL) of the Cell Counting Kit (CCK8, 40203ES60, Yeasen, Shanghai, China) was added to each well at a volume of 10 μL/well and allowed to react for a period of 30 min. Subsequently, dimethyl sulfoxide (DMSO) was introduced into each well followed by reading of absorbance using a microplate reader set at an wavelength of 450 nm.

### 4.6. Western Blot

Total protein was extracted from RAW264.7 macrophages and primary peritoneal macrophages using radio immunoprecipitation lysis buffer (RIPA, Solarbio, Beijing, China) supplemented with phenylmethylsulfonyl fluoride (PMSF) and phosphatase inhibitors (Solarbio, Beijing, China). The protein was collected, and its concentration was measured using a Pierce BCA protein assay kit (Thermo, Rockford, IL, USA). Equal amounts of proteins were separated by electrophoresis on SDS-PAGE gels and subsequently transferred onto PVDF membranes. Blocking was with TBST containing non-fat milk powder, the membranes were incubated overnight at 4 °C with primary antibodies. The primary antibodies against NRF2, p-AKT, AKT, iNOS (Cell Signaling Technology, Danvers, MA, USA), and HO-1, NQO-1, COX-2, p-GSK-3β, GSK-3β, p-AMPK-α1, AMPK-α1, p-ERK, EKR, p-JNK, JNK, p-P38, P38, p-NF-κB p65, NF-κB p65, and GAPDH (ABclonal, Wuhan, China) were diluted to a ratio of 1:2000. Before adding secondary antibodies (goat anti-rabbit or goat anti-mouse) diluted to a concentration of 1:10,000 (ABclonal, Wuhan, China), the membranes were washed four times with TBST for 15 min each time. Membranes were visualized using an Amersham Imager 600 (a gel imaging system from GE Co., Fairfield, CT, USA) after applying enhanced chemiluminescence. For detailed information of WB procedures, refer to our previous study [50].

### 4.7. Real-Time Quantitative PCR

Total RNA was extracted from cells/colon tissue using Trizol reagent (TransGen Biotech) following the manufacturer’s protocol. The isolated RNA was treated with DNase I (Sigma, St. Louis, MO, USA), quantified, and reverse transcribed into cDNA using TransScript first-strand cDNA synthesis SuperMix (TransGen Biotech, Beijing, China). Real-time quantitative PCR was performed in the ABI PRISM^®^7500 real-time PCR system (Applied Biosystems, Foster City, CA, USA) utilizing SYBR^®^ Premix Ex Taq™ II (Tli RNase H Plus) (TaKaRa, Dalian, China). The primer sequences are shown below. TNF-α, F: 5′-GCAACTGCTGCACGAAATC-3′, R: 5′-CTGCTTGTCCTCTGCCCAC-3′; IL-1β, F: 5′-GTTCCCATTAGACAACTGCACTACAG-3′,R: 5′-GTCGTTGCTTGGTTCTCCTTGTA-3′; IL-6, F: 5′-CCAGAAACCGCTATGAAGTTCC-3′, R: 5′-GTTGGGAGTGGTATCCTCTGTGA-3′; IL-9, F: 5′-ATGTTGGTGACATACATCCTTGC-3′, R: 5′-TGACGGTGGATCATCCTTCAG-3; IL-13, F: 5′-TGAGCAACATCACACAAGACC-3′, R: 5′-GGCCTTGCGGTTACAGAGG-3′; IL-23, F: 5′-CAGCAGCTCTCTCGGAATCTC-3′, R: 5′-TGGATACGGGGCACATTATTTTT-3′; β-actin, F: 5′-GTCAGGTCATCACTATCGGCAAT-3′, R: 5′-AGAGGTCTTTACGGATGTCAACGT-3′. Each of the samples were analyzed in triplicate, in a similar manner to our previous study [51].

### 4.8. Reactive Oxygen Species (ROS) Detection

RAW264.7 macrophages and primary peritoneal macrophages were cultured in 6-well plates and pre-treated with a concentration of 10 μM CEP for 1 h. Subsequently, the cells were exposed to LPS for a period of 12 h. Intracellular levels of ROS were quantified using the Reactive Oxygen Species Assay Kit (Solarbio, CA1410, Beijing, China) using a DCFH-DA ROS probe following the manufacturer’s instructions. The fluorescence intensity was then measured using flow cytometry and a fluorescence microscope. Detailed experimental procedures are outlined in the kit instructions.

### 4.9. MDA and Reduced GSH Detection

The MDA content in the serum of mice and primary peritoneal macrophages was quantified using a Malondialdehyde (MDA) Content Assay Kit (Solarbio, BC0025, Beijing, China). The GSH content in the serum of mice, RAW264.7 macrophages and primary peritoneal macrophages was determined using the Reduced Glutathione (GSH) Content Assay Kit (Solarbio, BC1175, Beijing, China). Detailed experimental procedures are outlined in the kit instructions.

### 4.10. Statistical Analysis

In this study, the data were presented as mean ± SEM. Statistical analysis was performed using GraphPad Prism software version 7.00. Group comparisons were conducted using one-way ANOVA followed by the least significant difference test (* *p* < 0.05, ** *p* < 0.01, *** *p* < 0.001). Two-group comparisons were analyzed using Student’s *t*-test (* *p* < 0.05). Each group in this study was replicated three times.

## 5. Conclusions

In conclusion, our study based on in vitro and in vivo data demonstrates that CEP exerts protective effects against oxidative stress and inflammatory responses by modulating the activation of the AMPK-α1/AKT/GSK-3β/NRF2 signaling pathway. Specifically, NRF2 plays a crucial role in safeguarding against oxidative stress and inflammatory response in LPS-induced macrophages and DSS-induced mice. Furthermore, CEP also triggers the phosphorylation of the JNK/ERK and NF-κB p65 signaling pathways, which are crucial for mediating inflammatory response. Our findings highlight the potential of CEP in ameliorating intestinal oxidative stress and inflammatory response. Moreover, the anti-oxidative and anti-inflammatory properties of CEP may have implications for other diseases characterized by inflammatory response and oxidative stress; however, further extensive experiments are warranted to validate this hypothesis. Nevertheless, this study substantiates the antioxidant and anti-inflammatory effects of CEP while elucidating its potential mechanisms of action. 

## Data Availability

All data are contained within the article.
